# The TRANSFER Approach for assessing the transferability of systematic review findings

**DOI:** 10.1186/s12874-019-0834-5

**Published:** 2020-01-17

**Authors:** Heather Munthe-Kaas, Heid Nøkleby, Simon Lewin, Claire Glenton

**Affiliations:** 1 Norwegian Institute of Public Health, Oslo, Norway; 2 Health Systems Research Unit, South African Medical Research Council, Cape Town, South Africa; 3 Cochrane Norway, Oslo, Norway

**Keywords:** Transferability, Applicability, Indirectness, Relevance, Evidence, Systematic review methodology, GRADE, GRADE-CERQual, Stakeholder engagement

## Abstract

**Background:**

Systematic reviews are a key input to health and social welfare decisions. Studies included in systematic reviews often vary with respect to contextual factors that may impact on how transferable review findings are to the review context. However, many review authors do not consider the transferability of review findings until the end of the review process, for example when assessing confidence in the evidence using GRADE or GRADE-CERQual. This paper describes the TRANSFER Approach, a novel approach for supporting collaboration between review authors and stakeholders from the beginning of the review process to systematically and transparently consider factors that may influence the transferability of systematic review findings.

**Methods:**

We developed the TRANSFER Approach in three stages: (1) discussions with stakeholders to identify current practices and needs regarding the use of methods to consider transferability, (2) systematic search for and mapping of 25 existing checklists related to transferability, and (3) using the results of stage two to develop a structured conversation format which was applied in three systematic review processes.

**Results:**

None of the identified existing checklists related to transferability provided detailed guidance for review authors on how to assess transferability in systematic reviews, in collaboration with decision makers. The content analysis uncovered seven categories of factors to consider when discussing transferability. We used these to develop a structured conversation guide for discussing potential transferability factors with stakeholders at the beginning of the review process. In response to feedback and trial and error, the TRANSFER Approach has developed, expanding beyond the initial conversation guide, and is now made up of seven stages which are described in this article.

**Conclusions:**

The TRANSFER Approach supports review authors in collaborating with decision makers to ensure an informed consideration, from the beginning of the review process, of the transferability of the review findings to the review context. Further testing of TRANSFER is needed.

## Background

Evidence-informed decision making has become a common ideal within healthcare, and increasingly also within social welfare. Consequently, systematic reviews of research evidence (sometimes called evidence syntheses) have become an expected basis for practice guidelines and policy decisions in these sectors. Methods for evidence synthesis have matured, and there is now an increasing focus on considering the transferability of evidence to end users’ settings (context) in order to make systematic reviews more useful in decision making [1–4]. End users can include individual, or groups of, decision makers who commission or use the findings from a systematic review, such as policymakers, health/welfare systems managers, and policy analysts [3]. The term stakeholders in this paper may also refer to potential stakeholders, or those individuals who have knowledge of, or experience with, the intervention being reviewed and whose input may be considered valuable where the review includes a wide range of contexts, not all of which are well understood by the review team.

Concerns regarding the interaction between context and the effect of interventions are not new: the realist approach to systematic reviews emerged in order to address this issue [5]. However, while there appears to be an increasing amount of interest, and literature, related to context and its role in systematic reviews, it has been noted that “the importance of context in principle has not yet been translated into widespread good practice” within systematic reviews [6]. Context has been defined in a number of different ways, with the common characteristic being a set of factors external to an intervention (but which may interact with the intervention) that may influence the effects of the intervention [6–9]. Within the TRANSFER Approach, and this paper, “context” refers to the multi-level environment (not just the physical setting) in which an intervention is developed, implemented and assessed: the circumstances that interact, influence and even modify the implementation of an intervention and its effects.

### Responding to an identified need from end users

We began this project in response to concerns from end users regarding the relevance of the systematic reviews they had commissioned from us. Many of our systematic reviews deal with questions within the field of social welfare and health systems policy and practice. Interventions in this area tend to be complex in a number of ways – for example, they may include multiple components and be context-dependent [10]. Commissioners have at times expressed frustration with reviews that (a) did not completely address the question in which they were originally interested, or (b) included few studies that came from seemingly very different settings. In one case, the commissioners wished to limit the review to only include primary studies from their own geographical area (Scandinavia) because of doubts regarding the relevance of studies coming from other settings despite the fact that there was no clear evidence that this intervention would have different effects across settings. Although we regularly engage in dialogue with stakeholders (including commissioners, decision makers, clients/patients) at the beginning of each review process, including a discussion of the review question and context, these discussions have varied in how structured and systematic they have been, and the degree to which they have influenced the final review question and inclusion criteria.

For the purpose of this paper, we will define stakeholders as anyone who has an interest in the findings from a systematic review, including client/patients, practitioners, policy/decision makers, commissioners of systematic reviews and other end users. Furthermore, we will define *transferability* as an assessment of the degree to which the context of the review question and the context of studies contributing data to the review finding differ according to a priori identified characteristics (transfer factors). This is similar to the definition proposed by Wang and colleagues (2006) whereby transferability is the extent to which the measured effectiveness of an applicable intervention could be achieved in another setting ([11] p. 77). Other terms related to transferability include applicability, generalizability, transportability and relevance and are discussed at length elsewhere [12–14].

### Context matters

Context is important for making decisions about the feasibility and acceptability of an intervention. Systematic reviews typically include studies from many contexts and then draw conclusions, for example about the effects of an intervention, based on the total body of evidence. When context – including that of both the contributing studies and the end user – is not considered, there can be serious, costly and potentially even fatal consequences.

The case of antenatal corticosteroids for women at risk of pre-term birth illustrates the importance of context: a Cochrane review published in 2006 concluded that “A single course of antenatal corticosteroids should be considered routine for preterm delivery with few exceptions” [15]. However, a large multi-site cluster randomized implementation trial looking at interventions to increase antenatal corticosteroid use in six low- and middle-income countries, and published in 2015, showed contrasting results. The trial found that: “Despite increased use of antenatal corticosteroids in low-birthweight infants in the intervention groups, neonatal mortality did not decrease in this group, and increased in the population overall” [16]. The trial authors concluded that “the beneficial effects of antenatal corticosteroids in preterm neonates seen in the efficacy trials when given in hospitals with newborn intensive care were not confirmed in our study in low-income and middle-income countries” and hypothesized that this could be due to, among other things, a lack of neonatal intensive care for the majority of preterm/small babies in the study settings [16]. While there are multiple possible explanations for these two contrasting conclusions (see Vogel 2017 [17];), the issue of context seems to be critical: “It seems reasonable to assume that the level of maternal and newborn care provided reflected the best available at the time the studies were conducted, including the accuracy of gestational age estimation for recruited women. Comparatively, no placebo-controlled efficacy trials of ACS have been conducted in low-income countries, where the rates of maternal and newborn mortality and morbidity are higher, and the level of health and human resources available to manage pregnant women and preterm infants substantially lower” [17]. The results from the Althabe (2015) trial highlighted that (in retrospect) the lack of efficacy trials of ACS from low-resource settings was a major limitation of the evidence base.

An updated version of the Cochrane review was published in 2017, and includes a discussion on the importance of context when interpreting the results: “The issue of generalisability of the current evidence has also been highlighted in the recent cluster-randomised trial (Althabe [2015]). This trial suggested harms from better compliance with antenatal corticosteroid administration in women at risk of delivering preterm in communities of low-resource settings” [18]. The WHO guidelines on interventions to improve preterm birth outcomes (2015) also include a number of issues to be considered before recommendations in the guideline are applied, that were developed by the Guideline Development Group and informed by both the Roberts (2006) review and the Althabe (2015) trial [19]. This example illustrates the importance of considering and discussing context when interpreting the findings of systematic reviews and using these findings to inform decision making.

### Considering context – current approaches

Studies included in a systematic review may vary considerably in terms of who was involved, where the studies took place and when they were conducted; or according to broader factors such as the political environment, organization of the health or social welfare system, or organization of the society or family. These factors may impact how transferable the studies are to the context specified in the review, and how transferable the review findings are to the end users’ context [20]. Transferability is often assessed by end users based on the information provided in a systematic review, and tools such as the one proposed by Schloemer and Schröeder-Bäck (2018) can assist them in doing so [21]. However, review authors can also assist in making such assessments by addressing issues related to context in a systematic review.

There are currently two main approaches for review authors to address issues related to context and the relevance of primary studies to a context specified in the review. One approach to responding to stakeholders’ questions about transferability is to highlight these concerns in the final review product or summaries of the review findings. Cochrane recommends that review authors “describe the relevance of the evidence to the review question” [22] in the review section entitled *Overall completeness and applicability of evidence*, which is written at the end of the review process. Consideration of issues related to applicability (transferability) is thus only done at a late stage of the review process. *SUPPORT summaries* are an example of a product intended to present summaries of review findings [23] and were originally designed to present the results of systematic reviews to decision makers in low and middle income countries. The summaries examine explicitly whether there are differences between the studies included in the review that is the focus of the summary and low- and middle-income settings [23]. These summaries have been received positively by decision makers, particularly this section on the relevance of the review findings [23]. In evaluations of other, similar products, such as Evidence Aid summaries for decision makers in emergency contexts, and evidence summaries created by The National Institute for Health and Care Excellence (NICE) [24–27], content related to context and applicability were reported as being especially valuable [28, 29].

While these products are useful, the authors of such review summaries would be better able to summarize issues related to context and applicability if these assessments were already present in the systematic review being summarized rather than needing to be made post hoc by the summary authors. However, many reviews often only include relatively superficial discussions of context, relevance or applicability, and do not present systematic assessments of how these factors could influence the transferability of findings.

There are potential challenges related to considering issues related to context and relevance *after* the review is finished, or even after the analysis is concluded. Firstly, if review authors have not considered factors related to context at the review protocol stage, they may not have defined potential subgroup analyses and explanatory factors which could be used to explain heterogeneity of results from a meta-analysis. Secondly, relevant contextual information that could inform the review authors’ discussion of relevance may not have been extracted from included primary studies. To date, though, there is little guidance for a review author on how to systematically or transparently consider applicability of the evidence to the review context [30]. Not surprisingly, a review of 98 systematic reviews showed that only one in ten review teams discussed the applicability of results [31].

The second approach, which also comes late in the review process, is to consider relevance as part of an overall assessment of confidence in review findings. The Grading of Recommendations Assessment, Development and Evaluation (GRADE) Approach for effectiveness evidence and the corresponding GRADE-CERQual approach for qualitative evidence [32, 33] both support review authors in making judgments about how confident they are that the review finding is “true” (GRADE: “the true effect lies within a particular range or on one side of a threshold”; GRADE-CERQual: “the review finding a reasonable representation of the phenomenon of interest” [33, 34]). GRADE and GRADE-CERQual involve an assessment of a number of domains or components, including methodological strengths and weaknesses of the evidence base, and heterogeneity or coherence, among others [32, 33]. However, the domain related to relevance of the evidence base to the review context (GRADE *indirectness* domain, GRADE-CERQual *relevance* component) appears to be of special concern for decision makers [3, 35]. Too often these assessments of indirectness or relevance that the review team makes may be relatively crude – for example, based on the age of participants or the countries where the studies were carried out, features that are usually easy to assess but not necessarily the most important. This may be due to a lack of guidance for review authors on which factors to consider and how to assess them.

Furthermore, many review authors only first begin to consider *indirectness* and *relevance* once the review findings have been developed. An earlier systematic and transparent consideration of transferability could influence many stages of the systematic review process and, in collaboration with stakeholders, could lead to a more thoughtful assessment of the GRADE *indirectness* domain and GRADE-CERQual *relevance* component. In Table 1 we describe a scenario where issues related to transferability are not adequately considered during the review process.

**Table 1:** The need for contextualizing evidence

*Scenario*: You are a systematic review author commissioned by a European government agency to conduct a review on the effectiveness of an intervention to reduce homelessness and improve number of days in stable housing for people who have been homeless. You identify, appraise and synthesise the evidence and use the GRADE approach to assess the certainty of the evidence, including an assessment of the directness of the evidence. At the end of the systematic review, you present the results to the commissioner and are faced with the criticism that the results will not transfer to their context because all of the included primary studies were conducted in the USA. Through multiple rounds of dialogue with stakeholders you discover that key contextual factors (for example, how long participants are homeless before participating in an intervention) are important to the success and viability of intervention in the end users’ context, and that the review has not considered these factors adequately. The end users therefore perceive the results of the review as not as useful to their decision making process as they anticipated.	

By engaging with stakeholders at an early stage of planning the review, review authors could ascertain what factors stakeholders judge to be important for their context and use this knowledge throughout the review process. Previous research indicates that decision makers’ perceptions of the relevance of the results and its applicability to policy facilitates the ultimate use of findings from a review [3, 23]. These decision makers explicitly stated that summaries of reviews should include sections on relevance, impact and applicability for decision making [3, 23]. Stakeholders are not the only source for identifying transferability factors, as other systematic reviews, implementation studies and qualitative studies may also provide relevant information regarding transferability of findings to specific contexts. However, this paper and the TRANSFER Approach focus on stakeholders specifically as it is our experience that stakeholders are often an underused resource for identifying and discussing transferability.

### Working toward collaboration

Involving stakeholders in systematic review processes has long been advocated by research institutions and stakeholders alike as a necessary step in producing relevant and timely systematic reviews [36–38]. Dialogue with stakeholders is key for (a) defining a clear review question, (b) developing a common understanding of, for instance, the population, intervention, comparison and outcomes of interest, (c) understanding the review context, and (d) increasing acceptance among stakeholders of evidence-informed practice and of systematic reviews as methods for producing evidence [38]. Stakeholders themselves have indicated that improved collaboration with researchers could facilitate the (increased) use of review findings in decision making [3]. However, in practice, few review teams actively seek collaboration with relevant stakeholders [39]. This could be due to time or resource constraints or access issues [40]. There is currently work underway looking at how to identify and engage relevant stakeholders in the systematic review process (for example, Haddaway 2017 [41];).

For those review teams who do seek collaboration, there is little guidance available on how to collaborate in a structured manner, and we are not aware of any guidance specifically focussed on considering transferability of review findings [42]. We are unaware of any guidance intended to support systematic review authors in considering transferability of review findings from the beginning of the review process (i.e. before the findings have been developed). The guidance that is available either focuses on a narrow subset of research questions (e.g. healthcare), is intended to be used at the end of a review process [12, 43], focuses on primary research rather than systematic reviews [44], or is theoretical in nature without any concrete stepwise guidance for review authors on how to consider and assess transferability [21]. Previous work has pointed out that stakeholders “need systematic and practically relevant knowledge on transferability. This may be supported through more practical tools, useful information about transferability, and close collaboration between research, policy, and practice” [21]. Other studies have also discussed the need for such practical tools, including more guidance for review authors that focuses on methods for (1) collaborating with end users to develop more precise and relevant review questions and identify a priori factors related to the transferability of review findings, and (2) systematically and transparently assessing the transferability of review findings to the review context, or a specific stakeholders’ context, as part of the review process [12, 45, 46].

The aim of the TRANSFER Approach is to support review authors in developing systematic reviews that are more useful for decision makers. TRANSFER provides guidance for review authors on how to consider and assess the transferability of review findings by collaborating with stakeholders to (a) define the review question, (b) identify factors a priori which may influence the transferability of review findings, and (c) define the characteristics of the context specified in the review with respect to the identified transferability factors.

## Aim

The aim of this paper is to describe the development and application of the TRANSFER Approach, a novel approach for supporting collaboration between review authors and stakeholders from the beginning of the review process to systematically and transparently consider factors that may influence the transferability of systematic review findings.

## Methods

We developed the TRANSFER Approach in three stages. In the first stage we held informal discussions with stakeholders to ascertain the usefulness of, guidance on assessing and considering the transferability of review findings. An email invitation to participate in a focus group discussion was sent to nine representatives from five Norwegian directorates that regularly commission systematic reviews from the Norwegian Institute of Public Health. In the email we described that the aim of the discussion would be to discuss the possible usefulness of a tool to assess applicability of systematic review findings to the Norwegian context. Four representatives attended the meeting from three directorates. The agenda for the discussion was a brief introduction to the terms and concepts, “transferability” and “applicability”, followed by an overview of the TRANSFER Approach as a method for addressing transferability and applicability. Finally we undertook an exercise to brainstorm transferability factors that may influence the transferability of a specific intervention to the Norwegian context. Participants provided verbal consent to participate in the discussion. We did not use a structured conversation guide. We took notes from the meeting, and collated the transferability issues that were discussed. We also collated responses regarding the usefulness of using time to discuss transferability with review authors during a project as simple yes or no responses (as well as any details provided with responses).

In the second stage we conducted a systematic mapping to uncover any existing checklists or other guidance for assessing the transferability of review findings, and conducted a content analysis of the identified checklists. We began by consulting systematic review authors in our network in March 2016 to get suggestions as to existing checklists or tools to assess transferability. In June 2016 we designed and conducted a systematic search of eight databases using search terms such as terms “transferability”, “applicability”, “generalizability”, etc. and “checklist”, “guideline”, “tool”, “criteria”, etc. We also conducted a grey literature search and searched the EQUATOR repository of checklists for relevant documents. Documents were included if they described a checklist or tool to assess transferability (or other related terms such as e.g., applicability, generalizability, etc.). We had no limitations related to publication type/status, language or date of publication. Documents that discussed transferability at a theoretical level or assessed the transferability of guidelines to local contexts were not included. The methods and results of this work are described in detail elsewhere (Munthe-Kaas H, Nøkleby H: The TRAN SFER Framework for assessing transferability of systematic review findings, forthcoming). The output from this stage was a list of transferability factors, which became the basis for the initial version of a ‘conversation guide’ for use with stakeholders in identifying and prioritizing factors related to transferability.

In the third stage, we undertook meetings with stakeholders to explore the use of a structured conversation guide (based on results of the second stage) to discuss the transferability of review findings. We used the draft guide in meetings with stakeholders in three separate systematic review processes. We became aware of redundancies in the conversation guide through these meetings, and also of confusing language in the conversation guide. Based on this feedback and our notes from these meetings we then revised the conversation guide. The result of this process was a refined conversation guide as well as guidance for review authors on how to improve collaboration with stakeholders to consider transferability, and guidance on how to assess and present assessments of transferability.

## Results

In this section we begin by presenting the results of the exploratory work around transferability, including the discussions with stakeholders, and experiences of using a structured conversation guide in meeting with stakeholders. We then present the TRANSFER Approach that we subsequently developed including the purpose of the TRANSFER Approach, how to use TRANSFER, and a worked example of TRANSFER in action.

### Findings of the exploratory work to develop the TRANSFER Approach

#### Discussions with stakeholders

The majority of the 3 h discussion with stakeholders was spent on the exercise. We described for participants a systematic review that had recently been commissioned (by one of the directorates represented) on the effect of supported employment interventions for disabled people on employment outcomes. The participants brainstormed the potential differences between the Norwegian context and other contexts and how these differences might influence how the review findings could be used in the Norwegian context. The participants identified a number of issues related to the population (e.g., proportion of immigrants, education level, etc.), the intervention (the length of the intervention, etc.), the social setting (e.g., work culture, union culture, rural versus urban, etc.) and the comparison interventions (e.g., components of interventions given as part of “usual services”). After the exercise was completed, the participants debriefed on the usefulness of such an approach for thinking about the transferability of review findings at the beginning of the review process, in a meeting setting with review authors. All participants agreed that the discussion was (a) useful, and (b) worth a 2 to 3 h meeting at the beginning of the review process. There was discussion regarding the terminology, however, related to transferability, specifically who is responsible for determining transferability. One participant felt that the “applicability” of review findings should be determined by stakeholders, including decision makers, while “transferability” was a question that can be assessed by review authors. There was no consensus among participants regarding the most appropriate terms to use. We believe that opinions expressed within this discussion may be related to language, for instance, how the Norwegian terms for ‘applicability’ and ‘transferability’ are used and interpreted. The main findings from the focus group discussion were that stakeholders considered meeting with review authors early in the review process to discuss transferability factors to be a good use of time and resources.

#### Systematic mapping and content analysis of existing checklists

We identified 25 existing checklists that assess transferability or related concepts. Only four of these were intended for use in the context of a systematic review [14, 43, 45, 47]. We did not identify any existing tools that covered our specific aims. Our analysis of the existing checklists identified seven overarching categories of factors related to transferability in the included checklists: population, intervention, implementation context (immediate), comparison condition, outcomes, environmental context, and researcher conduct [30]. The results of this mapping are reported elsewhere [30].

#### Using a structured conversation guide to discuss transferability

Both the review authors and stakeholders involved in the three systematic review processes where an early version of the conversation guide was piloted were favorable to the idea of using a structured approach to discussing transferability. The initial conversation guide that was used in meetings with the stakeholders was found to be too long and repetitive to use easily. The guide was subsequently refined to be shorter and to better reflect the natural patterns of discussion with stakeholders around a systematic review question (i.e. population, intervention, comparison, outcome).

### The TRANSFER Approach: purpose

**Fig. 1:**
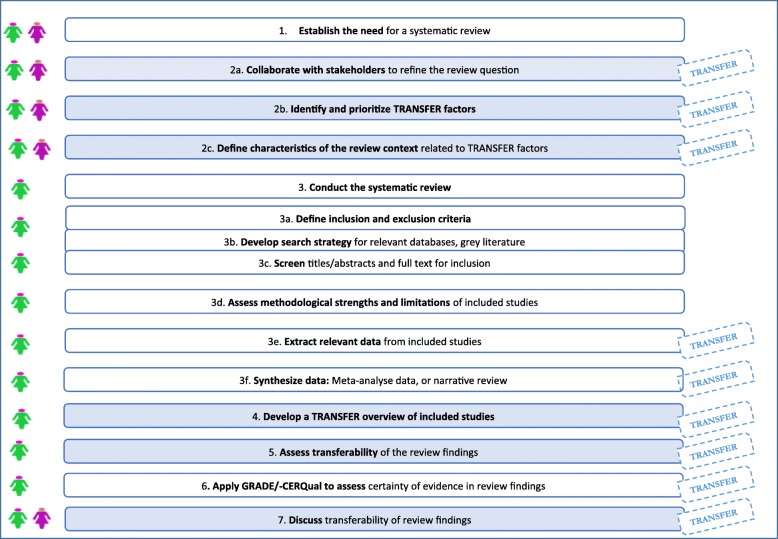
TRANSFER diagram

The exploratory work described above resulted in the TRANSFER Approach. The TRANSFER Approach aims to support review authors in systematically and transparently considering transferability of review findings from the beginning of the review process. It does this by providing review authors with structured guidance on how to collaborate with stakeholders to identify transferability factors, and how to assess the transferability of the review findings to the review context or other local contexts (see Fig. 1).

The TRANSFER Approach is intended for use in all types of reviews. However, as of now, it has only been tested in reviews of effectiveness related to population level interventions.

### How to use TRANSFER in a systematic review

The TRANSFER Approach is divided into seven stages that mirror the systematic review process. Table 2 outlines the stages of the TRANSFER Approach and the corresponding guidance and templates that support review authors in considering transferability at each stage (see Table 3). During these seven stages, review authors make use of the two main components of the TRANSFER Approach: (1) guidance for review authors on how to consider and assess transferability of review findings (including templates), and (2) a Conversation Guide to use with stakeholders in identifying and prioritizing factors related to transferability.

**Table 2:** What is new and what are the implications of the TRANSFER Approach?

*What is new?* This paper outlines new guidance for review authors on how to consider and assess the transferability of review findings to the context(s) specified in the review. The TRANSFER Approach supports review authors in making systematic and transparent assessments of transferability that can be used to provide a systematic and transparent assessment of the GRADE domain *indirectness* or the GRADE-CERQual component *relevance*. Close collaboration with decision makers is a key component of the TRANSFER Approach. *What is the implication of the TRANSFER Approach?* In many reviews, the review team only considers transferability (or related concepts such as applicability, indirectness, relevance) of the review findings at the end of the review process and in an ad hoc manner. By considering factors which may influence transferability early in the review process and in collaboration with decision makers, the review team is better able to systematically and transparently make an assessment of how these factors may influence the transferability of the review findings to the context specified in the review, or another context. Such systematic assessments of transferability will also provide transparency to assessments of the GRADE domain *indirectness* or GRADE-CERQual component *relevance*.

**Table 3:** TRANSFER Approach in the systematic review process – overview of relevant people and components involved in each stage

Relevant stages of the review process	People involved in each stage	Relevant TRANSFER component
1. Establish the need for a systematic review	Stakeholders Review authors	Not applicable
2. a. Collaborate with stakeholders to refine the review question b. Identify and prioritize TRANSFER factors c. Define the context specified in the review with respect to TRANSFER factors	Stakeholders Review authors	TRANSFER Guidance for review authors: a. PICO Template (Appendix 1) b. TRANSFER Conversation Guide (Appendix 2) c. TRANSFER Characteristics of context (Appendix 3)
3. Conduct the systematic review	Review authors	Not applicable
4. Compare the included studies to the context specified in the review (global and/or local) with respect to TRANSFER factors	Review authors	TRANSFER Guidance for review authors (TRANSFER table of included studies; Appendix 4)
5. Assess the transferability of the review findings to the context specified in the review (global and/or local)	Review authors	TRANSFER Guidance for review authors (TRANSFER assessment template; Appendix 5)
6. Apply GRADE for effectiveness or GRADE-CERQual to assess certainty/confidence in review findings	Review authors	TRANSFER Guidance for review authors (TRANSFER assessment template; Appendix 5)
7. Discuss transferability of review findings	Stakeholders Review authors	Not applicable

Once systematic review authors have gone through the seven stages outlined in Table 3, they come up with assessments of concern regarding each transferability factor. This assessment should be expressed as no, minor, moderate or serious concerns regarding the influence of each transferability factor for an individual review finding. This assessment is made for each individual review finding because TRANSFER assessments are intended to support GRADE/−CERQual assessments of indirectness /relevance, and the GRADE/−CERQual approaches require the review author to make assessments for each individual outcome (for effectiveness reviews) or review finding (for qualitative evidence syntheses). Assessments must be done for each review finding individually because assessments may vary across outcomes. One transferability factor may affect a number of review findings (e.g., years of experience of mentors in a mentoring program), in the same way that one risk of bias factor (e.g., selection bias as a consequence of inadequate concealment of allocations before assignment) may affect multiple review findings. However, it is also the case that one transferability factor can affect these review findings differently (e.g., average education level of the population may influence on finding and not another) in the same way that one risk of bias factor may affect review findings differently (e.g., detection bias, due to lack of blinding of outcome assessment, may be less important for objective finding, such as death). An overall TRANSFER assessment of transferability is then made by the review authors (also expressed as no, minor, moderate or serious concerns), based on the assessment(s) for each transferability factor(s). Review authors should then provide an explanation for the overall TRANSFER assessment and an indication of how each transferability factor may influence the finding (e.g. direction and/or size of the effect estimate). Guidance on making assessments is discussed in greater detail below. In this paper, we have, for simplicity, described transferability factors as individual and mutually exclusive constructs. Through our experience in applying TRANSFER, however, we have seen that transferability factors can influence and amplify each other. While the current paper does not address these potential interactions, other review authors will need to consider when transferability factors influence each other or when one factor amplifies the influence of another factor, for example, primary care health facilities in rural settings may both have both fewer resources and poorer access to referral centres, both of which may interact to negatively impact on health outcomes.

### TRANSFER in action

In the following section we present the stages of the TRANSFER Approach using a worked example. The scenario is based on a real review [48]. However, the TRANSFER Approach was not available when this review started and thus the conversation with decision makers was conducted post hoc. Furthermore, while the TRANSFER factors are those that the stakeholders identified, details related to both the review finding and the assessment of transferability were adapted for the purposes of this worked example in order to illustrate how TRANSFER could be applied to a review process.” The scenario focuses on a situation where a review is commissioned and the stakeholders’ context is known. In the case where the decision makers and/or their context is not well understood to the review team, the review team can still engage potential stakeholders with knowledge/experience related to the intervention being reviewed and the relevant contexts. .

#### Stage 1: Establish the need for a systematic review

Either stakeholders (in commissioning a review) or a review team (if initiating a review themselves) can establish the need for a systematic review (see example provided in Table 4). The process of defining the review question and context begins only after some need for a systematic review is established.

**Table 4:** Scenario – establishing the need for a systematic review

*Scenario*: The Norwegian State Housing Bank commissions your institution to undertake a systematic review on the effects of housing programmes on homelessness and housing stability. Specifically, the stakeholders want to know if providing free housing to a homeless individual with mental health problems in Norway will reduce the number of nights (s)he spends homeless and increase the number of nights (s)he spends in stable housing.	

#### Stage 2a: Collaborate with stakeholders to refine the review question

After defining the need for a systematic review, the review team, together with stakeholders need to meet to refine the review question (see example provided in Table 5). Part of this discussion will need to focus on establishing the type of review question being asked, and the corresponding review methodology that will be used (e.g., a review to examine intervention effectiveness or a qualitative evidence synthesis to examine barriers and facilitators to implementing an intervention). The group will then need to define the review question including, for example, the population, intervention, comparison and outcomes. A secondary objective of this discussion is to ensure common understanding of the review question, including how the systematic review is intended to be used. During this meeting the review team and stakeholders can discuss and agree upon, for example, the type of population and intervention(s) they are interested in, the comparison(s) they think are the most relevant, and the outcomes they think are the most important. By using a structured template to guide this discussion, the review team can be sure they cover all topics and questions in a systematic fashion. We have developed and used a basic template for reviews of intervention effectiveness that review authors can use to lead this type of discussion with stakeholders (see Appendix 1). Future work will involve adapting this template to different types of review questions and processes.

**Table 5:** Scenario – refining the review question

*Scenario*: You invite the review commissioners and a group of experts on homelessness to a meeting to refine the review question. You agree that the question they are asking concerns intervention effectiveness and that a systematic review of randomised trials is the most appropriate review type. Together you specify the population, intervention, comparison and outcomes of interest. The group agrees on the following review question: ‘What is the effect of housing programmes on homelessness and housing stability?’ The commissioners are interested in studies that include adult populations from any setting – the review question is therefore global in scope. However, the group defines a secondary question related to the transferability of the review findings to the Norwegian context. Thus you end up with a primary and secondary question: *• Primary review question: What is the effect of housing programmes on homelessness and housing stability?* *• Secondary review question: How do the results from this review transfer to the Norwegian context?*	

In some situations, such as in the example we provide, the scope of the review is broader (in this case, global) than the actual context specified in the review (in this case, Norway). The review may therefore include a broader set of interventions, population groups, or settings than the decision making context. Where the review scope is broader than the context specified in the review, a secondary review question can be added – for example, *How do the results from this review transfer to a pre-specified context?* Alternatively, where the context specified in the review context is the same as the end users’ context, such a secondary question would be unnecessary. When the review context or the local context is defined at a country level, the review authors and stakeholders will likely be aware of heterogeneity within that context (e.g., states, neighbourhoods, etc.). However, it is still often possible (and necessary) to ascertain and describe a national context. We need to further explore how decision makers apply review findings to the multitude of local contexts within, for example, their national context. Finally, in a global review initiated by a review team rather than commissioned for a specific context, a secondary question on the transferability of the review findings to a pre-specified context is unlikely to be needed.

#### Stage 2b. Identify and prioritize TRANSFER factors

In the scenario discussed in Table 6, stakeholders are invited to identify transferability factors through a structured discussion using the TRANSFER Conversation Guide (see Appendix 2). The identified factors are essentially hypotheses which need to be tested later in the review process. The aim of the type of consultation described above is to gather input from stakeholders regarding which contextual factors are believed to influence how/whether an intervention works. Where the review is initiated by the review team, the same process would be used, but with experts and people who are thought to represent stakeholders, rather than actual commissioners.

**Table 6:** Scenario – identifying TRANSFER factors

*Scenario*: During the meeting with commissioners, you use a structured conversation guide to identify and prioritize factors which may influence the transferability of the review findings to both the review context (global) and the local context specified in the secondary question (Norway). Together, you develop a number of hypotheses. However, the commissioners and expert group prioritize the following variables as potentially influencing transferability: (1) length of homelessness of participants at baseline; (2) the quality and comprehensiveness of usual housing services in the study context; and (3) climate (weather conditions) in the study context. The group agrees that these factors may influence the transferability of review findings – for example, individuals who have been homeless over longer periods of time are thought to respond less to interventions than those who have short, intermittent periods of homelessness. In addition, they hypothesise that an intervention may have a relatively smaller effect in a setting with high quality and comprehensive usual housing services compared to where the intervention is introduced in a setting with low quality usual services. Finally, they suggest that intervention participants in settings with a cold climate may consistently stay longer in stable housing when it is offered as part of an intervention due to climate rather than the intervention. The relationships between the above mentioned variables and the effect of the intervention are considered by the review authors to be hypotheses and treated as such. Following the meeting, you search for any evidence to support the hypotheses that the identified factors may influence transferability of the review findings. Evidence is found to support two of these hypotheses, and the third factor (climate) is included despite a lack of evidence supporting its influence on the effect of housing programmes. These factors are then listed in the protocol as explanatory factors on which subgroup analyses could be undertaken.	

The review authors may identify and use an existing logic model describing how the intervention under review works or another framework to initiate the discussion on transferability, for example to identify components of the intervention that could be especially susceptible to transferability factors or to highlight at what point in the course of the intervention transferability may become an issue [49, 50]. More work is needed to examine how logic models can be used at the beginning of the systematic review in order to identify potential transferability factors.

During this stage, the group may identify multiple transferability factors. However, we suggest that the review team, together with stakeholders, prioritize these factors and only include the most important three to five factors in order to keep data extraction and subgroup analyses manageable. Limiting the number of factors to be examined is based on our experience of piloting the framework in systematic reviews, as well as on guidance for conducting and reporting subgroup analyses [51]. Guidance on prioritizing transferability factors is still to be developed.

In accordance with guidance for conducting subgroup analyses in effectiveness reviews, the review team should search for evidence to support the hypotheses that these factors influence transferability, and indicate what effect they are hypothesised to have on the review outcomes [51]. We do not yet know how best to do this in an efficient way. To date, the search for evidence to support hypothetical transferability factors has involved a grey literature search of key terms related to the identified TRANSFER factors together with key terms related to the intervention, as well as searching Epistemonikos for qualitative systematic reviews on the intervention being studied. Other approaches, however, may include searching databases such as Epistemonikos for systematic reviews related to the hypotheses, and/or focused searches of databases of primary studies such as MEDLINE, EMBASE, etc. Assistance of an information specialist may be helpful in designing these searches and it may be possible to focus down on specific contexts, which would reduce the number of records that need to be searched. The efforts made will need to be calibrated to the resources available and the approach used should be described clearly to enhance transparency. In the case where no evidence is available for a transferability factor that stakeholders believe to be important, the review team will need to decide whether or not to include that transferability factor (depending, for example, on how many other factors have been identified), and provide justification for its inclusion in the protocol. The identified factors should be included in the review protocol as the basis for potential subgroup analyses. Such subgroup analyses will assist the review team in determining whether or not, or to what extent, differences with respect to the identified factor influence the effect of the intervention. This is discussed in more detail under Stage 4. In qualitative evidence syntheses, the review team may predefine subgroups according to transferability factors and contrast and compare perceptions/experiences/barriers/facilitators of different groups of participants according to the transferability factors.

#### Stage 2c: Define characteristics of the review context related to TRANSFER factors

In an intervention effectiveness review, the review context is typically defined in the review question according to inclusion criteria related to the population, intervention, comparison and outcomes (see example provided in Table 7). We recommend that this be extended to include the transferability factors identified in Stage 2, so that an assessment of transferability can be made later in the review process. If the review context does not include details related to the transferability factors, the review authors will be unable to assess whether or not the included studies are transferable to the review context. In this stage the review team works with the stakeholders to specify how the identified transferability factors manifest themselves in the context specified in the review (e.g., global context and Norwegian context).

**Table 7:** Scenario – defining characteristics of the review context related to TRANSFER factors

*Scenario:* The review authors in collaboration with the stakeholders go through the transferability factors identified in the previous stage (Stage 2b) and specify how the characteristics of the review context relate to these factors as follows:	
Context specified in the review: Global	
Transferability factors	Characteristics of review context
Average length of homelessness among homeless individuals	Length of time spent homeless by individuals included in the studies
Quality of usual housing services	Range of quality of usual housing services offered in various study settings
Climate (weather conditions)	Range of weather conditions (warm, cold, temperate climates, etc.) in the study settings
The review authors then specify the characteristics of the secondary context (Norway) according to the transferability factors identified. Research from Norway indicates that almost two thirds of the homeless population have been homeless for six months or longer [52]; usual housing services are of relatively high quality and comprehensive; and the cold season in Norway is sufficiently severe that this may be an important factor. Thus, you define the secondary context of interest as follows:	
Secondary context: Norway	
Transferability factors	Characteristics of specified context
Length of homelessness:	Most people who are homeless are homeless for more than 6 months at a time
Quality of usual services:	Residents who experience homelessness are provided high quality and comprehensive housing services as part of usual services
Climate:	Very cold winters

In cases where the review context is global, it may be challenging to specify characteristics of the global context for each transferability factor. In that case, the focus may be on assessing whether a sufficiently wide range of contexts are represented with respect to each transferability factor. Using the example above, the stakeholders and review team could decide that the transferability of the review findings would be strengthened if studies represented a range of usual housing services conditions in terms of quality and comprehensiveness, or if studies from both warm and cold climate settings are included.

#### Stage 3: conduct the systematic review

Several stages of the systematic review process may be influenced by discussions with stakeholders that took place in Stage 2 and the transferability factors that have been identified (see example in Table 8). These include defining the inclusion criteria, developing the search strategy and developing the data extraction form. In addition to standard data extraction fields, the review authors will need to extract data related to the identified transferability factors. This is done in a systematic manner where review authors also note where the information is not reported. For some transferability factors, such as environmental context, additional information may be identified through external sources. For other types of factors it may be necessary to contact study authors for further information.

**Table 8:** Scenario – conducting the systematic review

*Scenario:* You conduct the systematic review using standard methods for reviews of effectiveness. The identified transferability factors influence the search strategy (e.g. limits related to country, time, etc.) and inclusion criteria (e.g. limits related to population, setting for implementation of the intervention, etc.). Ten studies match your inclusion criteria, and you extract data from these using a standard form. In addition to the characteristics related to study design, population, intervention and outcomes, you also extract data related to length of homelessness at baseline, quality and comprehensiveness of usual housing services in the comparison condition, and the climate in the setting where the study is conducted. When a study does not include information related to the transferability factor, you record “not reported” for that study. In many of the studies, information regarding climate is not reported. You therefore identify documents external to the included studies that can give you information regarding climate for each study setting.	

#### Stage 4: compare the included studies to the context specified in the review (global and/or local) with respect to TRANSFER factors

This stage is about organizing the included studies according to their characteristics related to the identified transferability factors. The review authors should record these characteristics in a table – this makes it easy to get an overview of the contexts of the studies included in the review (see example in Table 9). There are many ways to organize and present such an overview. In the scenario above, the review authors created simple dichotomous subcategories for each transferability factor, which was related to the local context specified in the secondary review question.

**Table 9:** Scenario – Comparing the contexts of the included studies to the context specified in the review

*Scenario:* For each of the identified transferability factors you have selected, you record the characteristics of the included studies. You decide to use a dichotomous system in order to sort the studies more easily into subgroups related to the transferability factors: length of homelessness at baseline: <6 months, > 6 months; quality of usual housing services: high, low; climate: cold seasons, temperate (see Figure A below).			
*Figure A. TRANSFER overview of included studies*			
Studies/Factors	Length of homelessness	Quality of usual HOUSING services	Climate
Study 1	> 6 months	High quality	Cold
Study 2	< 6 months	High quality	Cold
Study 3	< 6 months	High quality	Cold
Study 4	> 6 months	High quality	Cold
Study 5	< 6 months	Low quality	Cold
Study 6	< 6 months	Low quality	Cold
Study 7	> 6 months	Low quality	Cold
Study 8	< 6 months	Low quality	Cold
Study 9	> 6 months	Low quality	Cold
Study 10	< 6 months	Low quality	Cold

#### Stage 5: assess the transferability of review findings

Review authors should assess the transferability of a review finding to the review context, and in some cases may also consider a local context (see example in Table 10). When a review context is global, the review team may have fewer concerns regarding transferability if the data come from studies from a range of contexts, and the results from the individual studies are consistent. If there is an aspect of context for which there is no evidence, this can be highlighted in the discussion.

**Table 10:**
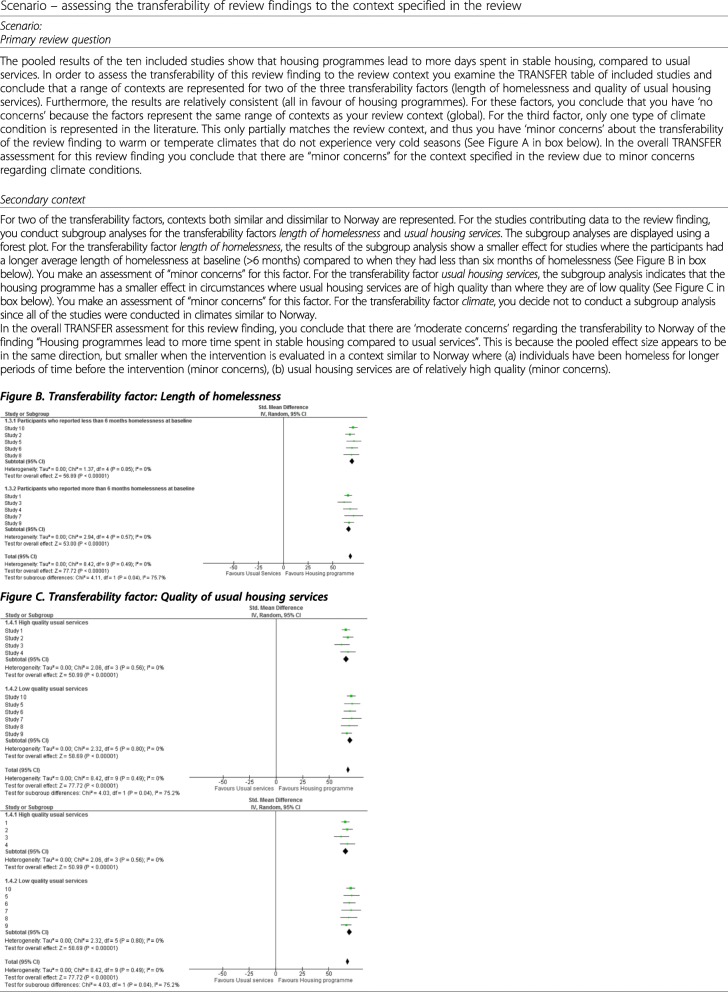
Scenario – assessing the transferability of review findings to the context specified in the review

In summary, when assessing transferability to a secondary context, the review team may:

1. Consider conducting a subgroup, or regression, analysis for each transferability factor to explore the extent to which this is likely to influence the transferability of the review finding. The review team should follow standards for conducting subgroup analyses [51, 53, 54].
2. Interpret the results of the subgroup or regression analysis for each transferability factor and record whether they have no, minor, moderate or serious concerns regarding the transferability of the review finding to the local context.
3. Make an overall assessment (no, minor, moderate or serious concerns) regarding the transferability of the review finding based on the concerns identified for each individual transferability factor. At the time of publication, we are developing more examples for review authors and guidance on how to make this overall assessment.

The overall TRANSFER assessment involves subjective judgements and it is therefore important for review authors to be consistent and transparent in how they make these assessments (see Appendix 4).

#### Stage 6: Apply GRADE for effectiveness or GRADE-CERQual to assess certainty/confidence in review findings

TRANSFER assessments can be used alone to present assessments of the transferability of a review finding in cases where the review authors have chosen not to assess certainty in the evidence. However, we propose that TRANSFER assessments can also be used to support *indirectness* assessments in GRADE (see example in Table 11). Similar to how the Risk of Bias tool or other critical appraisal tools support the assessment of *Risk of Bias* in GRADE, the TRANSFER Approach can be used to increase the transparency of judgements made for the *indirectness* domain [55]. The advantages to using the TRANSFER Approach to support this assessment are:

1. Factors that may influence transferability are carefully considered a priori, in collaboration with stakeholders;
2. The GRADE table is supported by a transparent and systematic assessment of these transferability factors for each outcome, and the evidence available for these;
3. Stakeholders in other contexts are able to clearly see the basis for the *indirectness* assessment, make an informed decision regarding whether the *indirectness* assessment would change for their context, and make their own assessment of transferability related to these factors. In some cases the transferability factors identified and assessed in the systematic review may differ from factors which may be considered important to other stakeholders adapting the review findings to their local context (e.g., in the scenario described above, stakeholders using the review findings in a low income, warmer country with a less comprehensive welfare system).

**Table 11:**
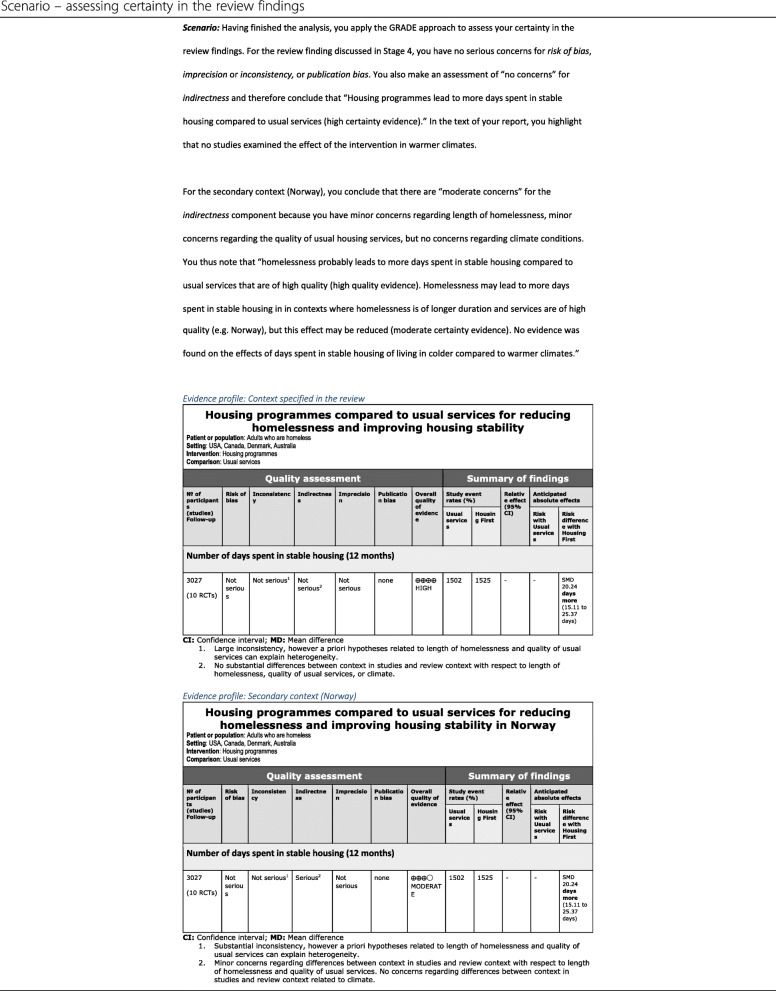
Scenario – assessing certainty in the review findings

Future work will be needed to develop methods of communicating the transferability assessment, how it is expressed in relation to a GRADE assessment and how to ensure that a clear distinction is made between TRANSFER assessments for a global context and, where relevant, a pre-specified local context.

#### Stage 7: Discuss transferability of review findings

In some instances it will be possible to discuss the transferability of the review findings with stakeholders prior to publication of the systematic review in order to ensure that the review team has adequately considered the TRANSFER factors as they relate to the context specified in the review (see example in Table 12). In many cases this will not be possible, and any input from stakeholders will be post-publication, if at all.

**Table 12:** Scenario – discussing transferability of the review findings

*Scenario:* Having finished the systematic review, you include in the report a discussion of the transferability of the review findings to the context specified in the review. You have the opportunity to share this section of the report with stakeholders prior to publication of the review. The stakeholders provide feedback on this, specifically on the adequacy of the discussion and that all relevant information has been included (the stakeholders’ do not have the opportunity to influence the findings of the review, only to give input on the discussion of transferability).	

## Discussion

To our knowledge, the TRANSFER Approach is the first attempt to consider the transferability of review findings to the context(s) specified in the review) in a systematic and transparent way from the beginning of the review process through to supporting assessments of certainty and confidence in the evidence for a review finding. Furthermore, it is the only known framework that gives clear guidance on how to collaborate with stakeholders to assess transferability. This guidance can be used in systematic reviews of effectiveness and qualitative evidence syntheses and could be applied to any kind of decision making [43].

The framework is under development and more user testing is needed to refine the conversation guide, transferability assessment methods, and presentation. Furthermore, it has not yet been applied in a qualitative evidence synthesis, and further guidance may be needed in order to support that process.

### Using TRANSFER in a systematic review

We have divided the framework into seven stages, and have provided guidance and templates for review authors for each stage. The first two stages are intended to support the development of the protocol, while stages three through seven are intended to be incorporated into the systematic review process.

The experience of review teams in the three reviews where TRANSFER has been applied (at the time this article is published) has uncovered potential challenges when applying TRANSFER. One challenge is related to reporting: the detail in which interventions, context and population characteristics are reported in primary studies is not always sufficient enough for the purpose of TRANSFER, as has been noted by others [56, 57]. With the availability of tools such as the TIDieR checklist and a number of CONSORT extensions, we hope that this improves and that the information that review authors seek is more readily available [58–60].

Our experience thus far has been that details concerning many of the TRANSFER factors prioritized by the stakeholders are not reported in the studies included in systematic reviews. In one systematic review on the effect of digital couples therapy compared to in-person therapy or no therapy, digital competence was identified as a TRANSFER factor [61]. The individual studies did not report this, so the review team examined national statistics for each of the studies included and reported this in the data extraction form [61]. The review team was unable to conduct a subgroup analysis for the TRANSFER factor. However, by comparing Norway’s national level of digital competence to that of the countries where the included studies were conducted, the authors were able to discuss transferability with respect to digital competence in the discussion section of the review [61]. They concluded that since the level of digital competence was similar in the countries of the included studies and Norway, the review authors had few concerns that this would be likely to influence the transferability of the review findings [61]. Without having identified this with stakeholders at the beginning of the process, there likely would have been no discussion of transferability, specifically the importance of digital competence in the population. Thus, even when it is not possible to do a subgroup analysis using TRANSFER factors, or even extract data related to these factors, the act of identifying these factors can contribute meaningfully to subsequent discussions of transferability.

### Using TRANSFER in a qualitative evidence synthesis

Although we have not yet used TRANSFER as part of a qualitative evidence synthesis, we believe that the process would be similar to that described above. The overall TRANSFER assessment could inform the GRADE-CERQual component *relevance*. A research agenda is in place to examine this further.

### TRANSFER for decision making

he TRANSFER Approach has two important potential impacts for stakeholders, especially decision makers: an assessment of transferability of review findings, and a close(r) collaboration review authors in refining the systematic review question and scope. A TRANSFER assessment provides stakeholders with (a) an overall assessment of the transferability of the review finding to the context(s) of interest in the review, and details regarding (b) whether and how the studies contributing data to the review finding differ from the context(s) of interest in the review, and (c) how any differences between the contexts of the included studies and the context(s) of interest in the review could influence the transferability of the review finding(s) to the context(s) of interest in the review (e.g. direction or size of effect). The TRANSFER assessment can also be used by stakeholders from other contexts to make an assessment of the transferability of the review findings to their own local context. Linked to this, TRANSFER assessments provide systematic and transparent support for assessments of the *indirectness* domain within GRADE and the *relevance* component within GRADE-CERQual. TRANSFER is a work in progress, and there are numerous avenues which need to be further investigated (see Table 13).

**Table 13:** TRANSFER in progress – priorities for further research

The TRANSFER Approach is still under development and some issues are still being discussed and piloted. Further research is needed to examine the following issues: - How do we systematically assess transferability for reviews that do not include a meta-analysis (e.g., where there is only a structured synthesis of the results in a narrative form?) [62] - What are the best methods for presenting assessments of transferability to different users? - Are factors identified by stakeholders in one setting likely to be important in another setting? How do we apply the findings from a review commissioned from decision makers in one context (such as hospitals in the Norwegian health system) to another decision making context (such as hospitals in one Spanish region)? - How can TRANSFER be used in the context of GRADE-CERQual (qualitative evidence syntheses), mixed methods reviews, and/or realist reviews? - How do review authors make an assessment of transferability where there are interactions between TRANSFER factors? - For a given systematic review using the TRANSFER Approach, what proportion of publications included in that systematic review include details related to the identified TRANSFER factors?	

The TRANSFER Approach also supports a closer collaboration between review authors and stakeholders early in the review process, which may result in more relevant and precise review questions, greater consideration of issues important to the decision maker, and better buy-in from stakeholders in the use of systematic reviews in evidence-based decision making [2].

## Conclusion

The TRANSFER Approach is intended to support review authors in collaborating with stakeholders to ensure that review questions are framed in a way that is most relevant for decision making and to systematically and transparently consider transferability of review findings. Many review authors already consider issues related to the transferability of findings, especially review authors applying the GRADE for effectiveness (*indirectness* domain) or GRADE-CERQual (*relevance* domain) approaches, and many review authors may engage with stakeholders. However current approaches to considering and assessing transferability appear to be ad hoc at best. Consequently, it often remains unclear to stakeholders how issues related to transferability were considered by review authors. By collaborating with stakeholders early in the systematic review process, reviews authors can ensure more precise and relevant review questions and an informed consideration of issues related to the transferability of the review findings. The TRANSFER Approach may therefore help to ensure that systematic reviews are relevant to and useful for decision making.

## Data Availability

The datasets used and/or analysed during the current study are available from the corresponding author on reasonable request.

## Appendix 1

**Table 14:** Sample PICO clarification template

	Suggested inclusion criteria	Questions for decision makers	Final inclusion criteria
Population	Everyone	Should we limit the population to only adults with families? Should we included participants with mental illness or substance abuse disorder?	Adults over 18 with/out families with/out mental illness/substance abuse disorders
Intervention/ Exposure	Housing programmes	Are there specific models that we are especially interested? Should we include housing programmes that include employment components? Are we interest in financial support only or programmes with case management?	Housing programmes with/out case management
Comparison intervention	Other / no intervention		Other / no intervention
Outcome	Days homeless, days in stable housing	Also include measurements related to quality of life? Health? Employment?	Primary: length of time homeless/in stable housing Secondary: QoL, health
Study design	RCTs	Should non-randomised studies be included?	RCTs
Other	English only, since 2000	What about other languages? Cut-off date for study inclusion?	All languages, all years

## Appendix 2

**Table 15:** TRANSFER Conversation Guide

TRANSFER Factor	Would you be concerned if data comes from contexts where…	Example	Notes
Environmental context			
Temporal context	… the data was collected at a different point in time?	e.g., studies conducted before 2000	
Geopolitical context	… the geographical, political or economic context is different?	e.g., studies conducted in post-conflict settings, settings where there is famine, high income settings, democratic settings, settings with colder/warmer temperatures, rural or urban settings	
Health or welfare system context	…the health or welfare system is arranged differently?	e.g., free versus fee-based primary health care, comprehensive vs. limited family welfare services	
Local professional/Expert opinion	… local professional/expert opinions are different?	e.g., experts are explicitly in favor or/against the intervention	
Community acceptability	… the local community has a different level/degree of acceptability for the intervention or the condition being addressed by the intervention?	e.g. religious reasons, ethical reasons, other social reasons	
Existence of alternative and/or co-existing interventions	… participants are exposed to alternative or supplemental interventions while participating in the intervention under examination?	e.g. contexts where all parents of small children are provided with free family counselling at the same time as they participate in a study where the intervention is online counselling for families with small children	
Participants			
Participant characteristics	…participants are different with respect to demographic characteristics, level of education, etc.?	e.g., studies on participants older/younger than those in your context, contexts with a different gender ratio,	
Participant compliance	…participants are different with respect to how well they follow instructions?	e.g., studies on pedestrian interventions to improve traffic safety in contexts where people are more/less likely to follow traffic rules	
Availability of personal support for participants	…participants have different access to personal support networks?	e.g., contexts where families live close by vs. individualistic cultures	
Characteristics of illness / condition and comorbidities	…participants’ condition or illness and comorbidities are different?	e.g., studies on premenstrual symptoms from Asian cultures versus western cultures where research suggests a difference in how women experience these conditions	
Participant acceptability and preferences	…participants level of acceptability and/or preferences regarding interventions/treatment, etc. are different?	e.g., studies of colon cancer screening interventions for men from contexts where they prefer to be called into/make their own annual appointments	
Participant need for / access to information	…participants have a different need for/access to/expectation of information?	e.g., studies from contexts where participants have a greater expectation of receiving comprehensive and detailed information regarding their treatment/intervention	
Intervention			
Details related to the intervention	… the intervention components/stages/phases/elements are routinely/consistently differ from your context?	These issues may be covered in while defining the review question and covered under inclusion/exclusion criteria in some cases.	
	…the intervention has a different duration, frequency, intensity?	These issues may be covered in while defining the review question and covered under inclusion/exclusion criteria in some cases.	
	…the intervention is delivered in a different setting?	These issues may be covered in while defining the review question and covered under inclusion/exclusion criteria in some cases.	
	…the availability and/or characteristics of materials/manuals for delivering the intervention is different?	These issues may be covered in while defining the review question and covered under inclusion/exclusion criteria in some cases.	
	…the intervention is delivered differently than it would be in a “real life setting”?	e.g. laboratory/efficacy studies	
	…the intervention has been tailored?	These issues may be covered in while defining the review question and covered under inclusion/exclusion criteria in some cases.	
	…the intervention is not delivered according to how it should be (i.e. implantation fidelity)?	e.g., the study authors describe clear deviations from how the intervention is intended to be developed (checklists such as TIDier could be helpful here)	
Category / status of the intervention	… the intervention is categorized differently?	e.g. policy, practice, programme, guideline	
Implementation of the intervention	…the intervention is delivered by service providers who differ from those in your setting?	e.g., number of service providers, characteristics of service providers, such as training or skill level or type/status of service providers’ position, their compliance with implementation directions, any other factors that may influence their motivation to implement the intervention, such as religious beliefs, cultural background or support from leadership/colleagues?	
	…the intervention is implemented by an organization that differs from those that would be expected to implement the intervention in your setting?	e.g., type of organization, size/structure, culture, policies, service and financing systems, interagency working relationships, available/allocated resources, communication/endorsement of intervention, evolution/sustainability of intervention	
Comparison intervention			
	…the quality or comprehensiveness of the comparison intervention is different?	This is likely to be important for the transferability of most interventions	
	…“usual services” is different with respect to quality, comprehensiveness or content?	This is likely to be important for the transferability of most interventions	
Outcomes			
	…the way an outcome is defined or measured is different, including length and intensity of follow-up?	e.g., culturally different scales to measure quality of life, long-term versus short-term follow-up	
	…the way an outcome is prioritized (by clients/patients) is different?	e.g., patient-important outcomes	

This is the most current version of the conversation guide and was developed based on feedback from review teams and stakeholders who used the previous tested version. Further testing of this version is planned

## Appendix 3

### TRANSFER characteristics of context

**Table 16:** TRANSFER Characteristics of review context

Context specifiedin the review: Global	
Transferability factors	Characteristics of review context
Average length of homelessness among homeless individuals	Length of time spent homeless by individuals included in the studies
Quality of usual housing services	Range of quality of usual housing services offered in various study settings
Climate (weather conditions)	Range of weather conditions (warm, cold, temperate climates, etc.) in the study settings

**Table 17:** TRANSFER Characteristics of secondary context

Secondary context: Norway	
Transferability factors	Characteristics of specified context
Length of homelessness:	Most people who are homeless are homeless for more than 6 months at a time
Quality of usual services:	Residents who experience homelessness are provided high quality and comprehensive housing services as part of usual services
Climate:	Very cold winters

## Appendix 4

### TRANSFER Table of Included Studies

**Table 18:** TRANSFER Table of Included Studies for review context

Review finding: Homeless programmes lead to fewer days spent homeless compared to usual services			
Context of interest: Context of interest in the review (Global) Average length of homelessness among homeless: varies Quality of usual housing services: varies Climate conditions: varies			
Factors / Studies	Length of homelessness	Quality of usual housing services	Climate conditions
Study 1	< 4 years	High quality	Cold
Study 2	< 2 years	High quality	Cold
Study 3	< 4 years	Low quality	Cold
Study 4	> 4 years	No services	Cold
Study 5	> 4 years	Low quality	Cold
Study 6	> 4 years	Low quality	Cold
SUMMARY	No concerns	No concerns	Minor concerns

**Table 19:** TRANSFER Table of Included Studies for secondary context

Review finding: Homeless programmes lead to fewer days spent homeless compared to usual services			
Context of interest: Norway Average length of homelessness among homeless: > 4 years Quality of usual housing services: high Climate conditions: Cold climate most of year			
Factors / Studies	Length of homelessness	Quality of usual housing services	Climate conditions
Study 1	< 4 years	High quality	Cold
Study 2	< 2 years	High quality	Cold
Study 3	< 4 years	Low quality	Cold
Study 4	> 4 years	No services	Cold
Study 5	> 4 years	Low quality	Cold
Study 6	> 4 years	Low quality	Cold
SUMMARY	Minor concerns	Minor concerns	No concerns

## Appendix 5

### TRANSFER assessment

**Table 20:** TRANSFER Assessment –context specified in the review

Review finding: Homeless programmes lead to fewer days spent homeless compared to usual services			
Context of interest: Global Average length of homelessness among homeless: varies Quality of usual housing services: varies Climate conditions: varies			
TRANSFER factors	Assessment	Explanation	Supporting studies
Length of homelessness of participants	No concerns	The studies represented a range of participants with length of homelessness at baseline ranging from 1 month to more than 6 months. All of the studies showed the same direction of effect.	1–10
Quality of «usual housing services»	No concerns	The studies represented a range of quality of usual services. All of the studies showed the same direction of effect.	1–10
Climate conditions	Minor concerns	The studies only partially represented the review context (cold climates). We are unsure if the finding is transferable to settings with warm or temperate climates.	1–10
Overall assessment	Minor concerns	There are no substantial differences between the included studies and the review context with respect to length of homelessness, quality of usual services or climate. However, the review finding is only based on evidence from cold climate settings, and we do not have any evidence available regarding how the intervention may work in warm settings.	1–10

**Table 21:** TRANSFER Assessment – secondary context

Review finding: Homeless programmes lead to fewer days spent homeless compared to usual services			
Secondary context: Norway Average length of homelessness among homeless: > 4 years Quality of usual housing services: high Climate conditions: Cold climate most of year			
TRANSFER factors	Assessment	Explanation	Supporting studies
Length of homelessness of participants	Minor concerns	The intervention appears to have a slightly reduced effect for individuals who have been homeless for longer than 6 months.	1–10
Quality of «usual housing services»	Minor concerns	The intervention appears to have a slightly reduced effect when compared with high quality usual services.	1–10
Climate conditions	No concerns	No concerns regarding differences between studies and review context related to climate.	1–10
Overall assessment	Moderate concerns	There are minor differences between the included studies and the review context with respect to length of homelessness, quality of usual services or climate. However, the review finding is only based on evidence from cold climate settings, and we do not have any evidence available regarding how the intervention may work in warm settings.	1–10

## Abbreviations

GRADE: The Grading of Recommendations Assessment, Development and Evaluation approach
GRADE-CERQual: The Confidence in Evidence from Reviews of Qualitative research approach
NICE: The National Institute for Health and Care Excellence
